# Development of machine learning models for predicting postoperative hyperglycemia in non-diabetic gastric cancer patients: a retrospective cohort study analysis

**DOI:** 10.3389/fendo.2025.1687745

**Published:** 2025-11-10

**Authors:** Nan Wang, Jie Zhang, Chaonan Fei, Ye Ding, Li Yang, Peibei Duan

**Affiliations:** 1 School of Nursing, Nanjing University of Chinese Medicine, Nanjing, China; 2 Department of Nursing, Jiangsu Province Hospital of Chinese Medicine, Affiliated Hospital of Nanjing University of Chinese Medicine, Nanjing, China; 3 Department of Oncology, Jiangsu Province Hospital of Chinese Medicine, Affiliated Hospital of Nanjing University of Chinese Medicine, Nanjing, China; 4 Department of Endocrinology, Jiangsu Province Hospital of Chinese Medicine, Affiliated Hospital of Nanjing University of Chinese Medicine, Nanjing, China

**Keywords:** postoperative hyperglycemia, non-diabetic patients, gastric cancer, machine learning, risk prediction, perioperative management, SHAP, SVM-radial

## Abstract

**Background:**

Postoperative hyperglycemia (POH) is a common metabolic complication in non-diabetic patients undergoing surgery for gastric cancer, and it significantly increases the risk of adverse outcomes. However, current prediction models primarily rely on a limited set of perioperative variables and conventional statistical methods, which often lack accuracy and generalizability. This study aimed to develop and validate a machine learning-based model for the early prediction of POH risk in non-diabetic patients following radical gastrectomy.

**Methods:**

This single-center, retrospective cohort study included 393 non-diabetic patients who underwent radical gastrectomy for gastric cancer between March 2021 and September 2024. A total of 38 perioperative clinical features covering preoperative, intraoperative, and early postoperative periods were collected. The primary outcome was POH, defined as a fasting venous plasma glucose level ≥ 7.8 mmol/L within 24 hours post-surgery. Nine machine learning algorithms, including Support Vector Machine with a radial basis function kernel (SVM-radial), Random Forest, XGBoost, and Logistic Regression, were developed and compared. Model performance was evaluated using accuracy, the area under the receiver operating characteristic curve (AUC), recall, and F1-score. Shapley Additive Explanations (SHAP) analysis was employed to interpret the model and identify key predictive factors.

**Results:**

The incidence of POH was 42.7%. Among all models, the SVM-radial model achieved the best test-set performance (AUC = 0.758, accuracy = 0.724, F1 = 0.743, recall = 0.750, Brier score = 0.186, calibration slope = 1.07).The model exhibited excellent discrimination, predictive accuracy, and probability calibration, indicating strong generalization capabilities and potential clinical utility. Seven key predictors were identified: operation duration, nutritional risk score, sex, surgical approach 2 (robotic surgery), preoperative fasting blood glucose, thrombosis risk score, and alkaline phosphatase. SHAP analysis confirmed the non-linear contributions of these features to POH risk and supported their interpretability for clinical decision-making.

**Conclusion:**

A novel machine learning-based model, utilizing multi-dimensional perioperative features, can accurately predict the risk of POH in non-diabetic patients with gastric cancer. The SVM-radial model demonstrated superior predictive performance and clinical interpretability, providing a viable tool for early risk stratification and personalized glycemic management in the surgical setting.

## 1 Introduction

Gastric cancer is the fifth most commonly diagnosed malignancy and the third leading cause of cancer-related death worldwide, with approximately 1.1 million new cases and 770,000 deaths annually, representing a major global public health challenge (1, 2). Epidemiological data indicate that Asia bears roughly 60% of the global burden, with China exhibiting the highest incidence and mortality rates (3). Despite advances in radical surgical techniques and widespread adoption of multidisciplinary treatment strategies (4), postoperative complications remain a critical determinant of both short- and long-term outcomes, with reported incidence rates of approximately 12% (5).

POH, a common metabolic response after radical gastrectomy, frequently occurs in patients without pre-existing diabetes and tends to be underestimated in clinical practice. Studies have reported that approximately 55.5% of non-diabetic gastric cancer patients experience postoperative blood glucose levels exceeding 126 mg/dL, with severe hyperglycemia (>200 mg/dL) occurring in 6.3% of cases (6). Compared with normoglycemic patients, those with POH demonstrate significantly higher complication rates (63.6% *vs*. 13%), as well as reduced 5-year overall survival (45% *vs*. 57%) and disease-free survival (46% *vs*. 68%) (7, 8). The underlying mechanisms involve stress-induced counter-regulatory hormone release, enhanced inflammatory responses, and insulin resistance (9, 10). Notably, non-diabetic patients with comparable hyperglycemia exhibit worse prognoses than diabetic counterparts, suggesting that POH may reflect more profound physiological dysregulation (11).

Current POH risk assessment tools primarily rely on traditional statistical models. For example, the postoperative complication model developed by Dong et al. (12) does not specifically target non-diabetic populations, while the logistic regression model by Wang et al. (13) is limited to preoperative and intraoperative variables, without incorporating critical postoperative factors. Most existing models employ linear regression (14, 15), which is inherently limited in capturing nonlinear relationships, variable interactions, and dynamic risk profiles. Furthermore, no standardized prediction tool tailored for POH in non-diabetic gastric cancer patients has been established, leading to reliance on empirical judgment in clinical practice and limiting the effectiveness of personalized interventions.

Recently, machine learning (ML) techniques have demonstrated superior performance over conventional statistical methods in early diagnosis and risk prediction (16). ML algorithms offer advantages such as high-dimensional data processing, nonlinear pattern recognition, and real-time predictive capability, and have been effectively applied to forecast perioperative complications including infection, thrombosis, and malnutrition (17–19). However, the application of ML for predicting POH in gastric cancer patients remains limited, with few high-performing and interpretable models specifically tailored to non-diabetic populations.

Considering the unique characteristics of Chinese gastric cancer patients—including distinct genetic backgrounds, dietary habits, and surgical practices—developing a locally adapted POH risk prediction model is of significant clinical relevance.

### 1.1 Study hypothesis

We hypothesize that machine learning algorithms can accurately predict POH by integrating multidimensional clinical data, outperforming traditional statistical approaches and identifying key risk factors. Based on this hypothesis, the present study aims to construct a POH risk prediction model for non-diabetic gastric cancer patients using multiple machine learning algorithms. By incorporating preoperative, intraoperative, and early postoperative variables, the model seeks to enable early identification, support precise clinical stratification and targeted interventions, and ultimately improve perioperative care and long-term patient outcomes.

## 2 Methods

### 2.1 Study design and patient selection

This was a single-center, retrospective cohort study based on real-world data. Eligible patients were non-diabetic individuals who underwent radical gastrectomy for gastric cancer at Jiangsu Province Hospital of Chinese Medicine between March 2021 and September 2024. The study protocol was approved by the Institutional Ethics Committee (approval date: April 14, 2025). Due to the retrospective nature of the study, the requirement for informed consent was waived.

Inclusion criteria were as follows:

1. Age ≥ 18 years;
2. Pre- or postoperative pathological diagnosis of gastric malignancy;
3. Underwent radical gastrectomy;
4. No prior history of diabetes mellitus.

Exclusion criteria included:

1. Patients were excluded if they met any of the following conditions;
2. had a history of other primary malignant tumors;
3. received preoperative medications affecting glucose metabolism (e.g., corticosteroids);
4. had missing key clinical data exceeding 20%.

**Figure 1:**
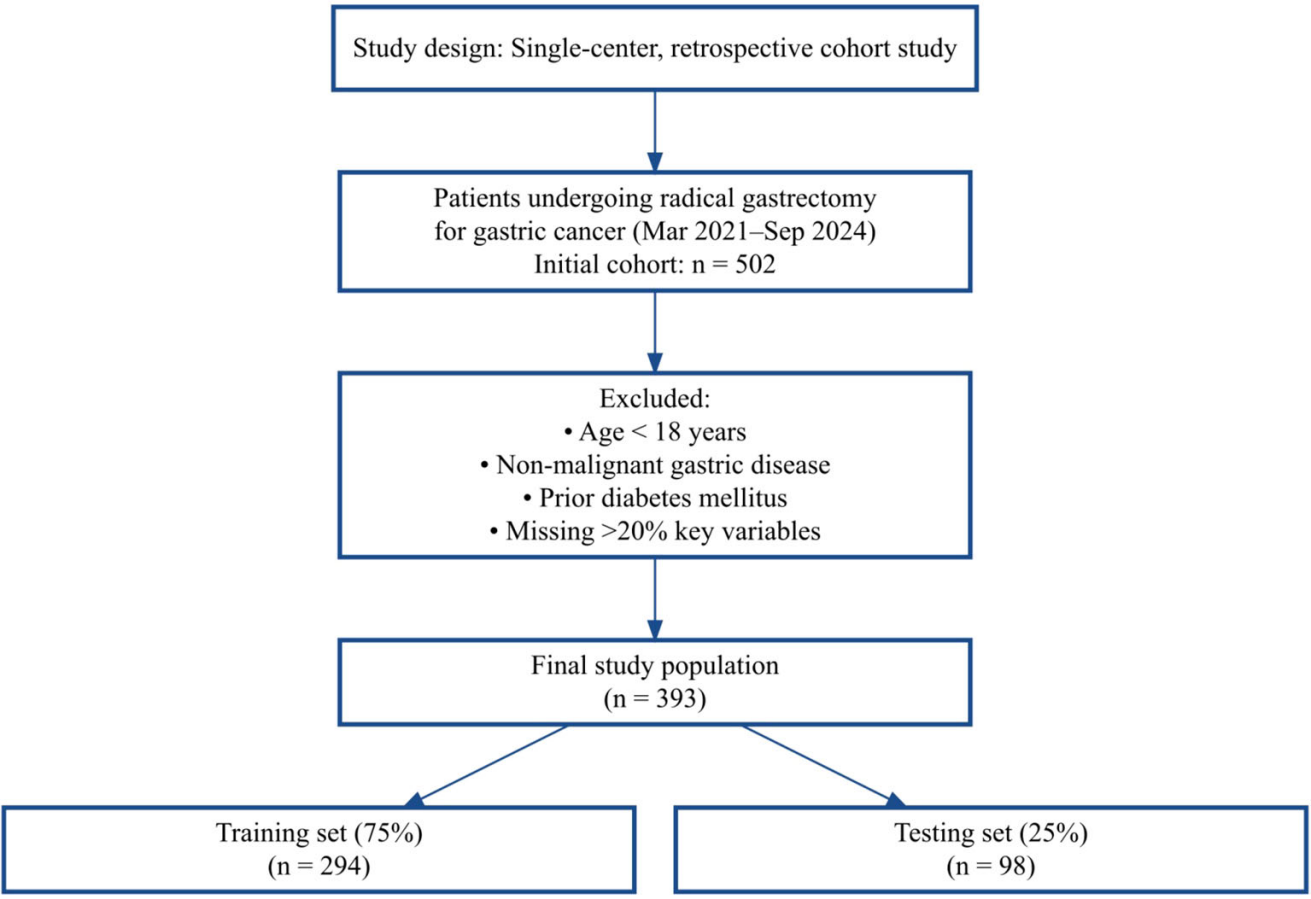
Flowchart of patient enrollment and inclusion/exclusion criteria.

As shown in **Figure 1**, 502 patients were screened, and after exclusions, 393 were included and randomly split into training and test sets.

### 2.2 Primary outcome

In accordance with established diagnostic criteria (10, 20), POH was defined as a fasting venous plasma glucose level ≥ 7.8 mmol/L within 24 hours post-surgery. This time point was selected based on prior evidence indicating that glucose levels typically peak within 24 hours after surgery (21). Venous plasma samples were used to minimize measurement bias compared to bedside glucose monitoring.

### 2.3 Data collection

Two trained researchers independently extracted patient data from the hospital’s electronic medical record (EMR) system. A double-entry method was used, and any discrepancies were resolved through discussion or adjudication by a third reviewer. A total of 38 demographic and clinical variables were collected.

#### 2.3.1 Demographic variables

Sex, age, body mass index (BMI), smoking status, alcohol consumption, family history of diabetes.

#### 2.3.2 Medical history

Hypertension, coronary artery disease, history of stroke, anemia (hemoglobin < 120 g/L for men, < 110 g/L for women), and hypoalbuminemia (albumin < 35 g/L).

#### 2.3.3 Preoperative laboratory data

White blood cell count, red blood cell count, hemoglobin, platelet count, neutrophil count, lymphocyte count, aspartate aminotransferase (AST), alanine aminotransferase (ALT), total protein, albumin, alkaline phosphatase (ALP), γ-glutamyl transferase (GGT), total bilirubin, direct bilirubin, fasting blood glucose(FBG), and carcinoembryonic antigen (CEA).

#### 2.3.4 Preoperative scores

Nutritional Risk Screening 2002 (NRS2002) and Padua Prediction Score (PPS) for venous thromboembolism (VTE) risk, hereafter referred to as the Thrombosis Risk Score.

#### 2.3.5 Surgical variables

Surgical Approach (laparoscopic, open, robotic), American Society of Anesthesiologists (ASA) classification, operative duration, intraoperative blood loss, fluid administration, and intraoperative use of corticosteroids and anesthetic agents (e.g., propofol, sufentanil, lidocaine).

#### 2.3.6 Postoperative variables

Nutritional support modality (enteral nutrition [EN], parenteral nutrition [PN], or a combination of EN and PN).

### 2.4 Missing data handling

To ensure data completeness, cases with more than 20% missing values in key variables were excluded. For the remaining cases, any record with missing data was removed, resulting in a final dataset of 393 patients for analysis. This approach ensured that all included cases had complete data, thereby avoiding any potential impact of missing values on the results.

### 2.5 Data preprocessing

Categorical variables, such as Surgical Approach, ASA classification, and type of nutritional support, were one-hot encoded; multi-label features, including intraoperative anesthetic use, were binarized; continuous variables were standardized using z-scores.

### 2.6 Feature selection and model development

#### 2.6.1 Statistical analysis

Given the large sample size in this study (n = 393) and based on the central limit theorem (CLT), continuous variables were assumed to be approximately normally distributed. Continuous variables are presented as mean ± standard deviation and compared using independent-samples z-tests. Categorical variables are presented as counts and percentages, and compared using χ² or Fisher’s exact tests, as appropriate. Relationships between continuous variables were assessed using Pearson correlation analysis.

#### 2.6.2 Data splitting

The dataset was randomly divided into a train set (75%) and a test set (25%) using stratified sampling to preserve the distribution of the outcome variable. The train set was used for feature selection, model train, and hyperparameter tuning, while the test set was reserved for the final model evaluation.

#### 2.6.3 Feature selection

Feature selection was first performed using LASSO logistic regression with L1 regularization (C = 0.1), retaining only variables with non-zero coefficients. These selected variables were then subjected to stepwise multivariate logistic regression, removing variables with P > 0.05 to determine the significant predictors. The coefficients of the variables selected by LASSO are presented in **Supplementary Figure S1**, providing a visual representation of each feature’s contribution to the model.

#### 2.6.4 Machine learning models

Using the final feature set, nine machine learning models were developed:

Logistic Regression (LR)

LASSO Logistic Regression (LR with LASSO)

k-Nearest Neighbors (KNN)

Linear Support Vector Machine (SVM linear)

the SVM-radial model

Decision Tree

Random Forest

Extreme Gradient Boosting (XGBoost)

Light Gradient Boosting Machine (LightGBM)

#### 2.6.5 Hyperparameter tuning

Hyperparameter optimization was performed using the Optuna framework, combining Bayesian optimization with 5-fold cross-validation. AUC was used as the primary performance metric to select the optimal hyperparameter set.

### 2.7 Model evaluation and interpretability analysis

Model performance was evaluated using AUC, accuracy, precision, recall, and F1 score. Receiver operating characteristic (ROC) curves and calibration plots were generated to assess model discrimination and calibration. Calibration performance was further quantified using the calibration slope, and Brier score. SHAP analysis was performed to quantify the contribution of each predictor to the model output, thereby enhancing model interpretability and clinical applicability.

All data processing and model development were conducted using Python version 3.11. All statistical tests were two-sided, and a P value ≤ 0.05 was considered statistically significant.

## 3 Results

### 3.1 Baseline characteristics

A total of 393 non-diabetic patients who underwent radical gastrectomy for gastric cancer were included in the study. Of these, 294 were allocated to the train set and 99 to the test set. The overall incidence of POH was 42.7% (168/393). As shown in **Table 1**, there were no statistically significant differences in demographic or baseline clinical characteristics between the train and test sets (all P > 0.05), indicating that the two cohorts were comparable.

**Table 1:** summarizes the baseline characteristics of the patients.

Feature	Train set (n = 294)	Test set (n = 98)	P value
Age, years	65.3 ± 11.0	64.7 ± 11.5	0.661
BMI, kg/m²	23.0 ± 3.3	23.1 ± 3.1	0.717
White blood cell count (WBC), ×10^9^/L	6.0 ± 3.0	5.9 ± 2.2	0.704
Red blood cell count (RBC), ×10¹²/L	4.0 ± 0.7	4.0 ± 0.6	0.854
Preoperative hemoglobin, g/L	115.8 ± 26.0	118.2 ± 20.8	0.412
Platelet count (PLT), ×10^9^/L	216.7 ± 87.1	212.5 ± 82.7	0.673
Neutrophil count (NEU), ×10^9^/L	3.9 ± 2.6	3.6 ± 2.0	0.341
Lymphocyte count (LYM), ×10^9^/L	1.4 ± 0.6	1.5 ± 0.5	0.422
AST, U/L	25.0 ± 32.2	22.0 ± 12.2	0.364
ALT, U/L	27.1 ± 67.8	20.6 ± 17.1	0.352
Total protein, g/L	82.1 ± 350.4	62.6 ± 6.5	0.583
Preoperative albumin, g/L	38.4 ± 4.4	38.2 ± 4.1	0.668
ALP, U/L	85.2 ± 38.8	85.5 ± 30.2	0.942
GGT, U/L	37.2 ± 118.3	34.6 ± 41.2	0.835
Total bilirubin, µmol/L	10.2 ± 10.2	9.6 ± 4.2	0.539
Direct bilirubin, µmol/L	2.7 ± 6.2	2.2 ± 1.0	0.400
Indirect bilirubin, µmol/L	7.5 ± 5.2	7.4 ± 3.6	0.838
Preoperative FBG, mmol/L	5.0 ± 1.0	5.1 ± 1.5	0.317
CEA, ng/mL	7.4 ± 38.2	11.5 ± 51.9	0.411
Operation duration, minutes	188.9 ± 62.3	188.9 ± 61.1	0.992
Intraoperative blood loss, mL	149.8 ± 163.4	131.7 ± 127.2	0.318
Intraoperative infusion volume, mL	1942.2 ± 699.9	1987.6 ± 682.2	0.576
Sex, n (%)			0.847
0 = Male	208 (71)	71 (72)	
1 = Female	86 (29)	27 (28)	
Alcohol history, n (%)			1.000
0 = No	247 (84)	82 (84)	
1 = Yes	47 (16)	16 (16)	
Smoking history, n (%)			0.940
0 = No	240 (82)	81 (83)	
1 = Yes	54 (18)	17 (17)	
Family history of diabetes, n (%)			0.478
0 = No	287 (98)	94 (96)	
1 = Yes	7 (2)	4 (4)	
Hypertension, n (%)			1.000
0 = No	188 (64)	62 (63)	
1 = Yes	106 (36)	36 (37)	
Coronary artery disease, n (%)			0.905
0 = No	276 (94)	91 (93)	
1 = Yes	18 (6)	7 (7)	
History of cerebrovascular disease, n (%)			0.661
0 = No	270 (92)	92 (94)	
1 = Yes	24 (8)	6 (6)	
Anemia, n (%)			0.201
0 = No	184 (63)	69 (70)	
1 = Yes	110 (37)	29 (30)	
Hypoproteinemia, n (%)			0.524
0 = No	233 (79)	74 (76)	
1 = Yes	61 (21)	24 (24)	
Surgical Approach, n (%)			0.707
0 = Laparoscopy	169 (57)	55 (56)	
1 = Open surgery	111 (38)	40 (41)	
2 = Robotic surgery	14 (5)	3 (3)	
ASA classification, n (%)			0.852
0 = Class I	1 (0)	0 (0)	
1 = Class II	161 (55)	56 (57)	
2 = Class III	131 (45)	42 (43)	
3 = Class IV	1 (0)	0 (0)	
Intraoperative corticosteroid use, n (%)			0.505
0 = No	33 (11)	8 (8)	
1 = Yes	261 (89)	90 (92)	
Anesthetic drugs, n (%)			0.803
0 = Propofol	14 (5)	7 (7)	
0 + 1 = Propofol + Sufentanil	3 (1)	0 (0)	
0 + 1+2 = Propofol + Sufentanil + Lidocaine	11 (4)	3 (3)	
0 + 2 = Propofol + Lidocaine	263 (89)	87 (89)	
1 = Sufentanil	1 (0)	0 (0)	
2 = Lidocaine	2 (1)	1 (1)	
Nutritional support, n (%)			1.000
0 = PN	289 (98)	97 (99)	
2 = EN+PN	5 (2)	1 (1)	
Nutritional risk screening 2002 (NRS2002), n (%)			0.840
Score 0	4 (1)	0 (0)	
Score 1	158 (54)	57 (58)	
Score 2	85 (29)	25 (26)	
Score 3	20 (7)	7 (7)	
Score 4	16 (5)	6 (6)	
Score 5	11 (4)	3 (3)	
Thromboembolism risk score (Padua score), n (%)			0.802
Score 0	3 (1)	0 (0)	
Score 1	4 (1)	1 (1)	
Score 2	7 (2)	2 (2)	
Score 3	48 (16)	14 (14)	
Score 4	80 (27)	35 (36)	
Score 5	87 (30)	28 (29)	
Score 6	51 (17)	15 (15)	
Score 7	14 (5)	3 (3)	

### 3.2 Feature selection and model construction

From the initial 38 candidate variables, seven potential predictors were identified through a combination of LASSO logistic regression and multivariable analysis: operation duration, preoperative fasting blood glucose, nutritional risk score, Sex, thrombosis risk score, ALP, and Surgical Approach 2 (robotic surgery).

To quantify the independent association of these variables with POH, a multivariable logistic regression analysis was performed. The results showed that operation duration (OR: 1.011, 95% CI: 1.006–1.016, P < 0.001), preoperative fasting blood glucose (OR: 1.328, 95% CI: 1.011–1.745, P = 0.042), nutritional risk score (OR: 1.373, 95% CI: 1.070–1.762, P = 0.013), Sex (OR: 2.518, 95% CI: 1.399–4.532, P = 0.002), thrombosis risk score (OR: 1.289, 95% CI: 1.048–1.585, P = 0.016), ALP (OR: 1.010, 95% CI: 1.001–1.019, P = 0.036), and Surgical Approach 2(Robotic surgery) (OR: 0.166, 95% CI: 0.039–0.709, P = 0.015) were independently associated with the occurrence of POH.

### 3.3 Nomogram construction and evaluation

**Figure 2:**
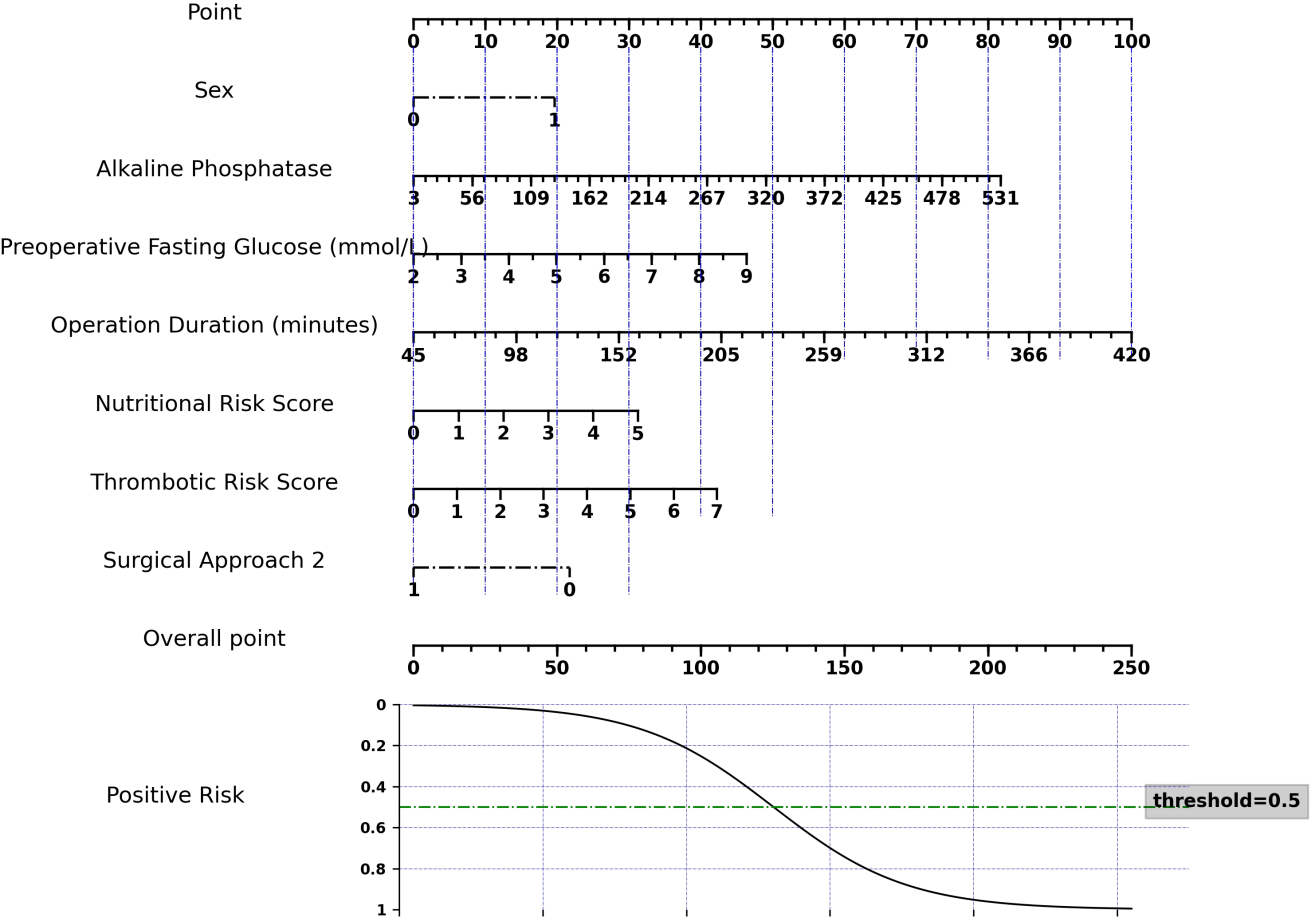
Nomogram for predicting the risk of POH risk in non-diabetic gastric cancer patients.

To facilitate clinical application of our logistic regression model, we developed a nomogram (**Figure 2**). This nomogram is not a new model, but a visual tool that translates complex regression coefficients into an intuitive scoring system. Each predictor is assigned points proportional to its regression coefficient, reflecting its relative impact on the model output. Clinicians sum the points to obtain a total score, which is then mapped to the predicted risk probability, corresponding exactly to the model’s output. This mapping ensures that the nomogram preserves the quantitative relationships between the regression coefficients and the machine learning model predictions, allowing accurate individualized risk assessment.

The model showed robust performance: AUC = 0.734 (train set) and 0.728 (test set), Brier score = 0.192, and calibration slope = 1.22, indicating good agreement between predicted and observed outcomes. Decision curve analysis confirmed that the nomogram provides significant clinical net benefit across a wide range of threshold probabilities (**Supplementary Figure S2**).

### 3.4 Machine learning model performance analysis

**Figure 3:**
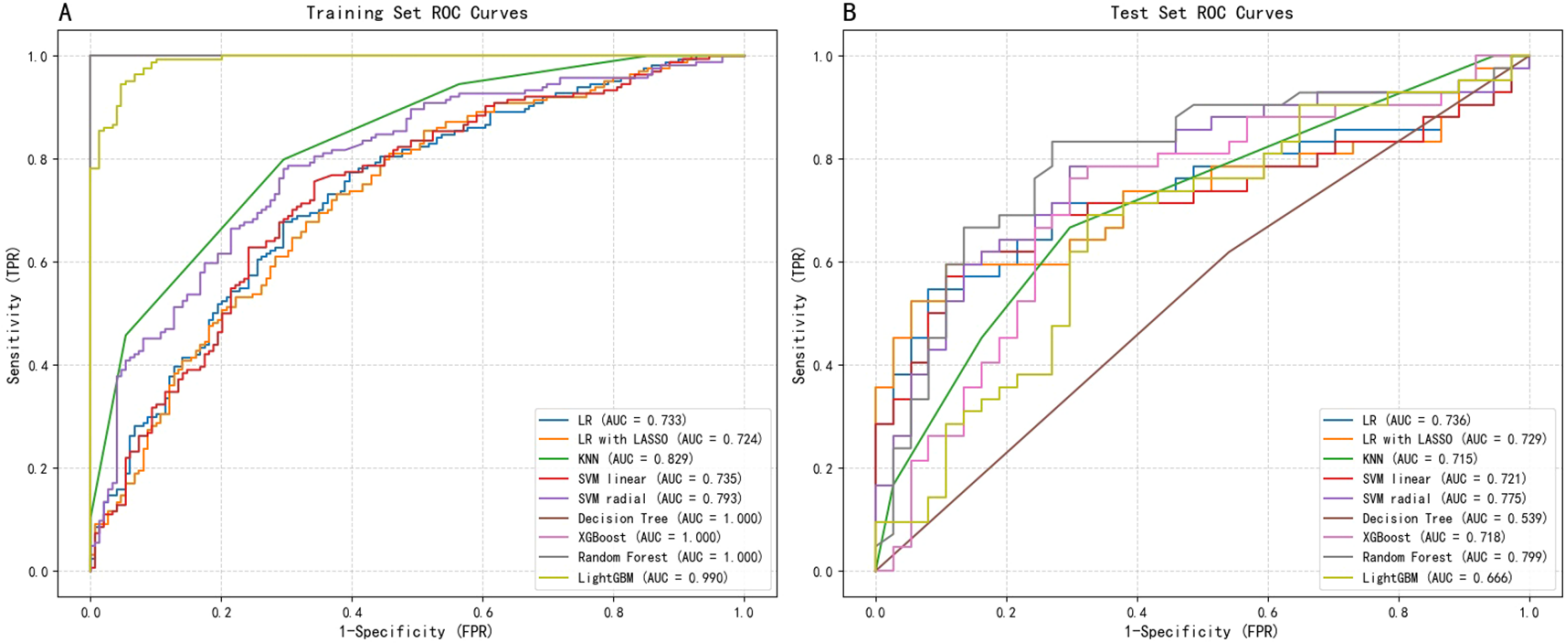
ROC curves for machine learning models on **(A)** train set and **(B)** test set.

A comprehensive comparison of the performance across nine machine learning models is presented in **Table 2**. Overall, ensemble algorithms based on trees demonstrated outstanding performance on the train dataset, with Random Forest, XGBoost, and Decision Tree models all achieving perfect AUC (1.0), while LightGBM attained an impressive AUC of 0.987. However, such near-perfect train set performance often implies overfitting risks—models may have learned specific noise within the data rather than generalizable patterns. **Figure 3** displays the ROC curves for all nine ML models on both the training and test datasets, providing a visual comparison of their discriminative performance and potential overfitting patterns.

**Table 2:** Performance comparison of different machine learning models in train and test datasets.

Model	Train AUC	Train accuracy	Train F1	Train recall	Test AUC	Test accuracy	Test F1	Test recall	Brier score	Calibration slope	*P*-value (SVM-radial *vs* Model)
SVM-radial	0.802	0.735	0.761	0.805	0.758	0.724	0.743	0.75	0.186	1.07	—
Logistic Regression	0.734	0.667	0.686	0.695	0.728	0.714	0.731	0.731	0.192	1.22	0.593
Random Forest	1	1	1	1	0.727	0.684	0.705	0.712	0.212	1.945	0.668
SVM linear	0.736	0.697	0.719	0.74	0.716	0.694	0.717	0.731	0.215	1.848	0.433
LR with LASSO	0.721	0.653	0.662	0.649	0.715	0.673	0.673	0.635	0.218	1.699	0.429
KNN	0.825	0.738	0.754	0.766	0.709	0.643	0.646	0.615	0.223	1.784	0.516
XGBoost	1	1	1	1	0.644	0.643	0.673	0.692	0.273	1.947	0.071
LightGBM	0.987	0.942	0.945	0.948	0.627	0.582	0.617	0.635	0.26	2.089	0.024
Decision Tree	1	1	1	1	0.579	0.582	0.61	0.615	0.418	0.551	0.008

To address this, we further strengthened internal validation, focusing on model stability and generalization capability. Taking the SVM-radial basis function model as an example, we conducted 10-fold cross-validation on the train set. Results revealed consistent performance across folds, with an average AUC of 0.737 ± 0.074, average accuracy of 0.717 ± 0.063, and average recall of 0.811 ± 0.124. This stable and balanced performance indicates the SVM model strikes a favorable equilibrium between fitting capability and generalization ability, thereby alleviating concerns about overfitting.

However, the true test of the model lies in the independent test set. test results revealed that the SVM-radial basis function model demonstrated the strongest generalization ability, achieving the highest AUC (0.758) and exhibiting robust discriminatory power. Conversely, models that performed perfectly on the train set (such as the decision tree, with a test AUC of 0.579) showed a significant decline in performance on the test set, once again reflecting their overfitting issues.

In clinical predictive applications, recall serves as a critical metric for evaluating a model’s ability to identify all high-risk patients. As shown in **Table 2**, the SVM-radial basis function model also achieved the highest recall (0.75) on the test set, particularly crucial in early-stage risk screening scenarios emphasizing high sensitivity.

Finally, we employed the DeLong test to compare the AUC differences between each model and the SVM-radial basis function model (column of **Table 2**). Results indicate no statistically significant difference between the SVM model and other high-performing models (e.g., Logistic Regression, p=0.593). Considering the SVM model’s highest AUC and recall on the test set alongside its stable cross-validation performance, it was ultimately selected as the optimal predictive model. This choice achieves the best equilibrium between discriminative capability and sensitivity towards high-risk patients.

### 3.5 Model explainability analysis

**Figure 4:**
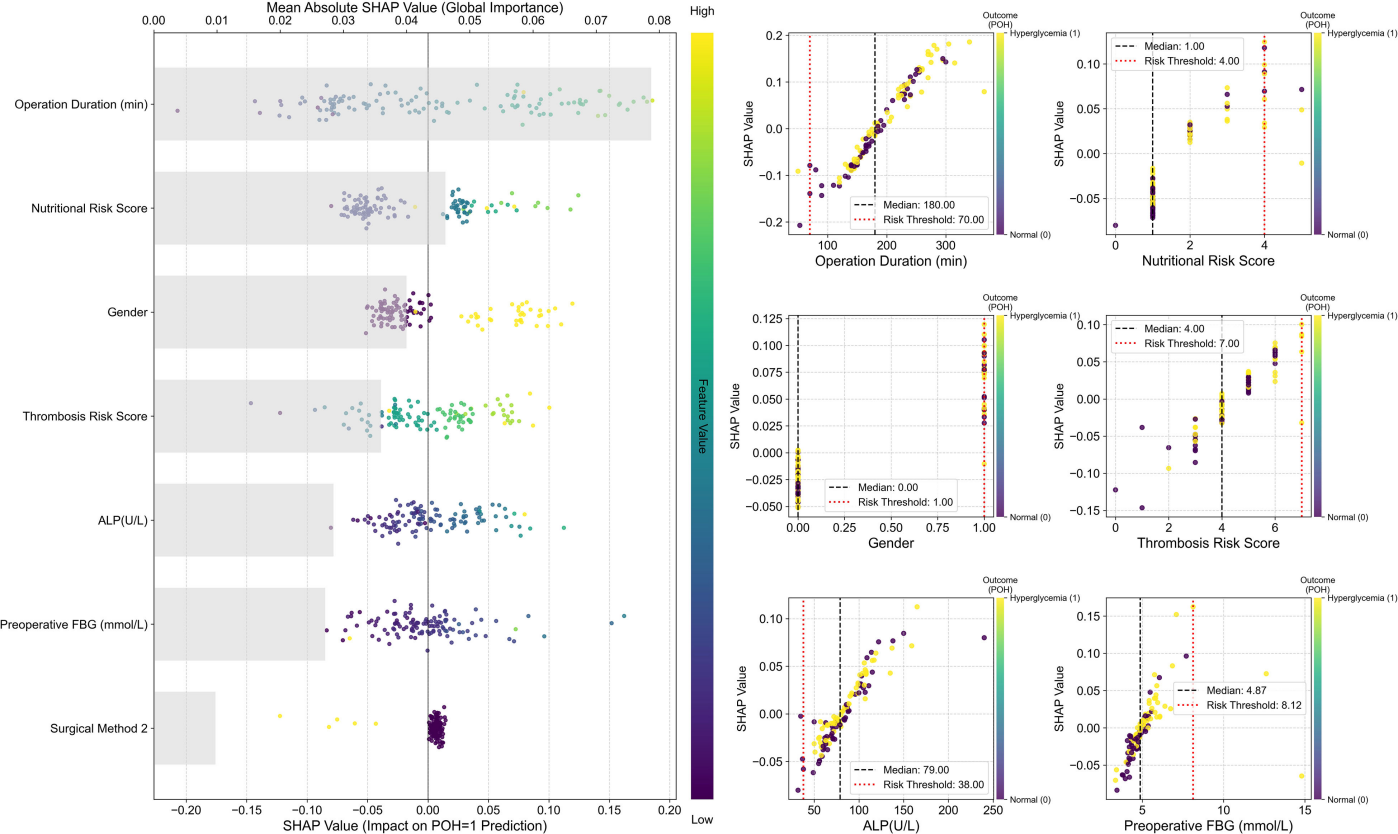
SHAP interpretability analysis of the optimal SVM model. Left: SHAP Summary Plot. The vertical axis ranks all predictors from highest to lowest global importance (mean absolute SHAP value); the horizontal axis displays SHAP values, quantifying each feature’s contribution to each prediction. Positive values indicate a push towards Postoperative hyperglycemia (POH = 1), while negative values indicate a push towards normal blood glucose. The color of each point indicates the magnitude of the corresponding feature value in that sample (defined by the color bar on the right: yellow/green denotes high values, purple/blue denotes low values). Right: SHAP Dependence Plots. Each subplot displays the raw feature value on the x-axis and its SHAP value on the y-axis. The color of each point indicates the sample’s actual outcome: yellow denotes hyperglycemia (Outcome=1), purple denotes no hyperglycemia (Outcome=0).

To conduct an in-depth analysis and validate the internal decision-making mechanisms of the optimal SVM model, we employed the SHAP framework for interpretability analysis (**Figure 4**).

The global feature importance plot (SHAP Summary Plot) on the left indicates that Operation Duration exerts the most significant influence on model predictions, followed sequentially by Nutritional Risk Score, Sex, Thrombosis Risk Score, ALP, and FBG. The dependency plot indicates that high-value samples (yellowish color) correspond to positive SHAP values for these features, suggesting that longer operation duration, higher nutritional and thrombosis risk scores, and elevated ALP and preoperative blood glucose levels significantly increase the predicted risk of POH. Conversely, SHAP values for Surgical Approach 2 cluster in the negative region, suggesting a potential protective effect within the model.

The SHAP Dependence Plots on the right further reveal local patterns and clinical threshold characteristics in the model’s predictions. Results indicate that POH risk does not increase linearly but undergoes abrupt changes at critical thresholds: SHAP values rise significantly when operating time exceeds 180 minutes or nutritional/thrombotic risk scores reach ≥4 points. Additionally, the model identified a risk threshold range for preoperative fasting blood glucose: risk begins to increase when FBG > 4.87 mmol/L and rises sharply beyond 8.12 mmol/L.

Overall, SHAP analysis validated the model’s predictive logic as highly consistent with clinical understanding, while quantifying the intensity and thresholds of key risk factors. This significantly enhances the model’s interpretability and clinical utility.

### 3.6 SVM model decision curve and risk stratification

**Figure 5:**
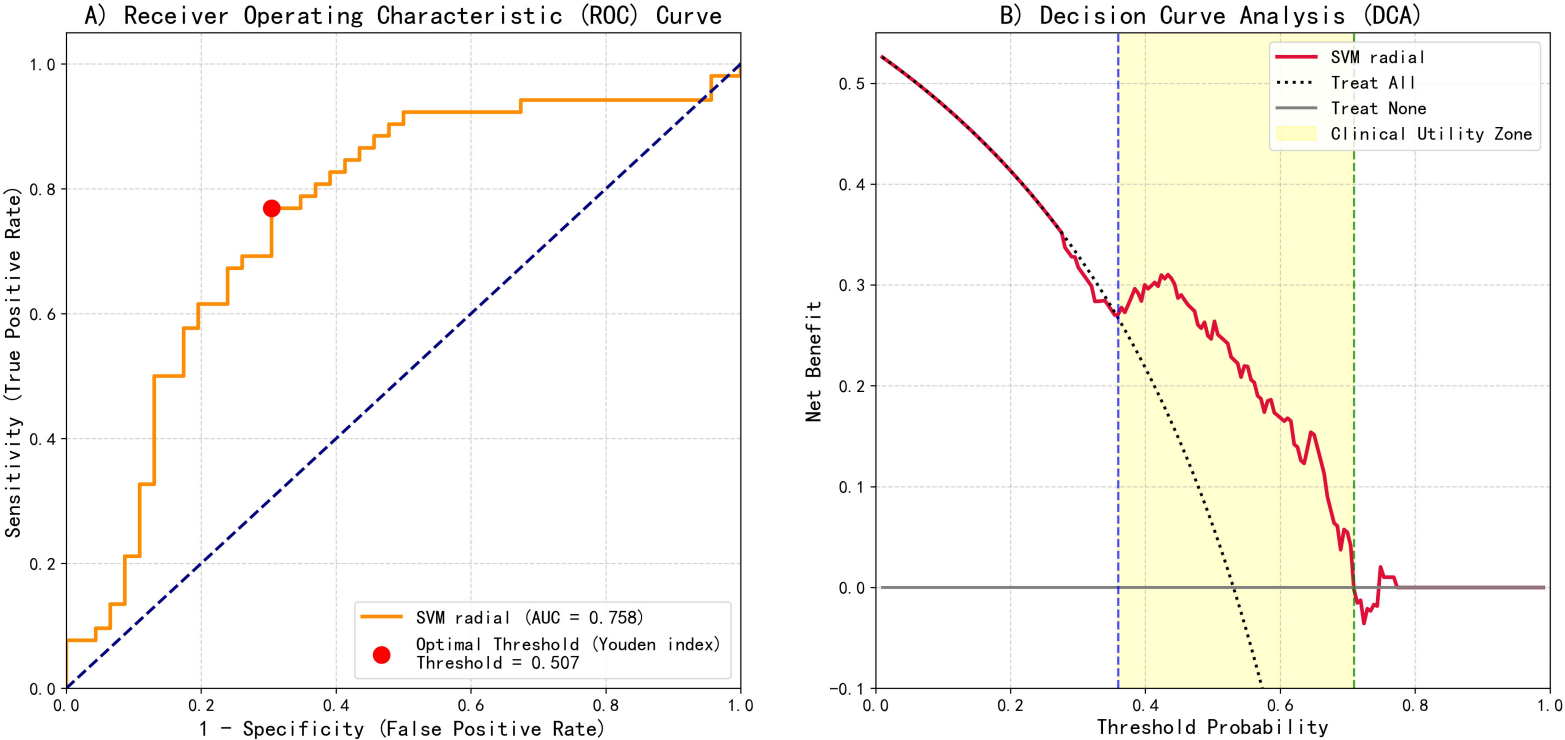
SVM-radial basis function model predictive performance evaluation: ROC and DCA. **(A)** ROC curve showing the optimal threshold (Youden index = 0.507) and AUC = 0.758. **(B)** DCA illustrating clinical net benefit compared with "treat-all" and "treat-none" strategies.

To translate the SVM-radial model’s predictions into a clinically actionable tool, we developed a risk stratification system based on the model’s performance on the test set. ROC curves analysis demonstrated the model’s favorable discriminatory capability, with an AUC of 0.758 (**Figure 5A**). The optimal statistical threshold determined by Youden’s index was a predicted probability of 0.507.

Furthermore, decision curve analysis (DCA) was conducted to assess clinical net benefit (**Figure 5B**). DCA results indicated positive clinical net benefit within the threshold probability range of 0.36 to 0.71.

Based on the aforementioned analysis, a three-tier risk stratification was established: low risk (< 0.30), intermediate risk (0.30–0.60), and high risk (≥ 0.60).

## 4 Discussion

In this study, we constructed and validated a machine learning model for predicting POH based on real-world clinical data from 393 non-diabetic patients undergoing radical gastrectomy. Among nine machine learning algorithms, the SVM-radial model demonstrated the highest predictive performance. SHAP interpretability analysis identified seven key predictors: operative duration, nutritional risk score (NRS), Sex, Surgical Approach type 2, preoperative fasting blood glucose, thrombotic risk score, and ALP.

POH was defined in this study as fasting blood glucose ≥ 7.8 mmol/L within 24 hours postoperatively. This definition may underestimate transient hyperglycemia induced by intraoperative or postoperative stress responses, thereby introducing potential misclassification bias. Fasting blood glucose was selected due to its standardizable measurement, high clinical feasibility, and relative independence from other perioperative confounders. Future studies may consider incorporating continuous or point-of-care glucose monitoring to more comprehensively reflect intraoperative and immediate postoperative glycemic dynamics (22).

To our knowledge, this represents the first study applying machine learning methods to predict POH risk in non-diabetic gastric cancer patients. By integrating 38 perioperative features—encompassing baseline characteristics, physiological indicators, surgical variables, and early postoperative data—we constructed a dynamic, multidimensional predictive framework capable of capturing the complexity of POH pathogenesis, surpassing traditional logistic regression models (23). Furthermore, we compared nine machine learning algorithms and optimized model parameters through five-fold cross-validation, ensuring scientific rigor and model stability. Concurrently, we subjected the optimal model to performance test via ten-fold cross-validation (10-fold CV) to validate its robustness and generalization capability. This aligns with recent literature indicating machine learning methods outperform traditional logistic regression in modelling perioperative complications such as surgical infections, acute kidney injury, and sepsis (17–19). By focusing on predicting POH risk in non-diabetic gastric cancer patients, this study complements and extends existing machine learning-based risk prediction frameworks.

On the test set, the SVM model achieved a high recall (0.75) while maintaining solid AUC (0.758), F1 score (0.743), and accuracy (0.724). In clinical practice, a high recall is particularly important, as it maximizes the identification of high-risk POH patients and reduces the likelihood of missed diagnoses (24). The nonlinear mapping capability of SVM-RBF enables it to capture complex feature interactions more accurately, thereby improving the discrimination of critical patients. By contrast, although the random forest showed perfect performance on the training set (AUC = 1.0, F1 = 1.0, recall=1.0), its performance on the test set dropped noticeably (AUC = 0.727, F1 = 0.705, recall=0.712), suggesting overfitting. While Miet et al. reported strong performance of random forest in predicting sepsis-associated severe acute kidney injury (sAKI), this method still exhibited overfitting in our dataset (25), indicating that model performance can be influenced by dataset-specific characteristics and clinical context, and should be evaluated accordingly. Therefore, in this clinical setting, recall is a more appropriate metric for model selection than AUC or F1, since the clinical consequences of missing high-risk patients far outweigh those of misclassifying low-risk patients. Selecting SVM as the optimal model based on test set recall is both reasonable and ensures sensitive identification of high-risk patients while maintaining overall predictive performance.

To translate SVM model predictive performance into a clinically actionable tool, we propose a three-tier risk management strategy based on predicted probabilities and Decision Curve Analysis (DCA) results (26). Decision curve analysis (DCA) showed that the clinically effective range of the model was approximately 0.36–0.71; however, to facilitate clinical memorization and ease of implementation, thereby improving operational feasibility, and to better balance sensitivity and specificity, thereby ensuring patient safety, we selected 0.30 and 0.60 as the risk stratification thresholds.

Low risk (<0.30): Within this probability range, the clinical net benefit of model-guided interventions is limited; thus, conventional management strategies represent a reasonable resource allocation choice.

Moderate risk (0.30–0.60): This core range demonstrates significant clinical net benefit over baseline strategies and encompasses the optimal Youden index threshold (0.507). Extended monitoring periods for closer observation are recommended.

High risk (≥0.60): Patients within this range exhibit substantially elevated event probability, necessitating enhanced clinical surveillance and intensive postoperative glucose monitoring.

This model-based, clinically optimized risk stratification method facilitates the allocation of limited healthcare resources towards medium-to-high-risk patients. It constitutes a clinically quantifiable and actionable risk management framework, whose potential for improving resource allocation efficiency has been validated in other clinical settings (27).

Among all predictors, duration of surgery represents a significant independent risk factor for POH (28). Prolonged surgical stress elevates catecholamine levels, intensifies gluconeogenesis and glycogenolysis, and induces pro-inflammatory cytokine release. These mechanisms collectively exacerbate insulin resistance and glycemic dysregulation (29, 30). Perioperative malnutrition constitutes an intervenable risk factor associated with adverse surgical outcomes (31). The positive SHAP distribution of NRS scores indicates that malnourished patients are more susceptible to stress-induced metabolic disorders, potentially linked to impaired β-cell function and reduced peripheral glucose utilization (32). Previous studies have also highlighted that malnutrition increases the risk of postoperative complications and is independently associated with poorer survival rates in cancer patients (33). The findings of this study further support the necessity of preoperative nutritional screening and intervention.

Sex differences also exerted a significant influence, consistent with prior studies (34): male patients exhibited approximately double the risk of POH compared to females. This may relate to the protective role of estrogen in glucose metabolism (35), though the underlying mechanisms warrant further investigation.

Preoperative fasting blood glucose serves as a strong predictor of POH, reflecting underlying metabolic stress and insufficient β-cell reserve prior to surgery, thereby elevating the risk of postoperative metabolic imbalance (36, 37). Additionally, thrombotic risk scores and ALP levels demonstrated significant predictive value. Elevated thrombotic risk scores not only indicate a hypercoagulable state but may also reflect systemic inflammation and activation of the coagulation axis. This pro-inflammatory state may prompt immune cells to release substantial inflammatory mediators, such as tumor necrosis factor-α (TNF-α). These mediators could inhibit downstream signaling of insulin receptor substrate-1 (IRS-1) by activating serine kinases including JNK and IKK, leading to impaired glucose utilization in peripheral tissues—insulin resistance. Concurrently, ALP is a recognized clinical marker for hepatic or skeletal disorders. As the central organ for glucose metabolism, impaired liver function may disrupt regulation of gluconeogenesis and glycogenolysis, thereby destabilizing blood glucose homeostasis. This suggests that thrombotic risk and ALP levels may interact via inflammatory-metabolic pathways to increase the risk of POH (38–40).

Notably, Surgical Approach 2 (robotic surgery) exhibited a negative SHAP distribution in the model, suggesting it may confer a protective effect. This finding may relate to the reduced trauma and lighter intraoperative stress associated with robotic surgery. Existing research indicates that robotic surgery results in less intraoperative blood loss and lower postoperative levels of C-reactive protein (CRP), a sensitive inflammatory marker linked to cardiovascular disease. CRP also exhibits a negative correlation with insulin sensitivity. It may therefore be inferred that robotic surgery, by reducing postoperative inflammation, could be associated with diminished insulin resistance, thereby facilitating the maintenance and restoration of postoperative metabolic homeostasis. Future studies could further compare the effects of different Surgical Approaches on perioperative metabolic homeostasis, providing guidance for optimizing surgical technique selection (41, 42).

Growing evidence indicates that POH in non-diabetic patients is independently associated with increased postoperative complication risk (43) and may adversely affect recovery by promoting metabolic and inflammatory dysregulation (44, 45). Nevertheless, this phenomenon is frequently overlooked in clinical practice, with postoperative glucose monitoring in non-diabetic patients remaining markedly inadequate (6). Early identification of POH risk facilitates the implementation of preventive interventions, enhances metabolic stress tolerance, and promotes postoperative recovery. Recognizing modifiable risk factors—such as nutritional status and surgery-related parameters—also provides a basis for risk prevention and control at the public health level.

This study has several limitations. First, as a single-center retrospective study with a limited sample size (n = 393) and fasting glucose measured only at 24 hours postoperatively, the generalizability of the findings may be limited. In addition, the model is applicable only to non-diabetic gastric cancer patients and lacks external validation; although internal stability was assessed through train-test splitting and 10-fold cross-validation, this may still be insufficient to ensure its applicability across different hospitals, regions, and ethnic populations. Similar machine learning studies predicting surgical complications also emphasize the necessity of external validation to confirm model utility across diverse populations (46).Therefore, future studies should perform external validation in multicenter cohorts encompassing diverse regions and ethnic groups to evaluate the model’s broader generalizability and performance.

Moreover, the model has the potential to be implemented as an online calculator or mobile application, enabling clinicians to assess postoperative hyperglycemia risk in real time based on preoperative patient characteristics. Integration with hospital information systems or electronic medical records could facilitate automated risk evaluation. Finally, the application of the model could inform clinical workflows, such as determining the intensity of postoperative glucose monitoring and the timing or dosing of insulin intervention, thereby optimizing perioperative management and improving patient outcomes.

## 5 Conclusion

This study successfully developed the first machine learning-based prediction model for POH in non-diabetic gastric cancer patients. The SVM-radial algorithm demonstrated superior predictive performance and identified seven critical risk factors, offering new insights into the pathogenesis of POH. The proposed model may facilitate early risk stratification and individualized management strategies, ultimately improving perioperative care and long-term outcomes.

## Data Availability

The datasets presented in this article are not readily available because The data and materials generated in this study are available from the corresponding author upon reasonable request. Requests must be reviewed and approved by the Ethics Committee of Nanjing University of Chinese Medicine, after which the corresponding author will provide the relevant data and materials. Requests to access the datasets should be directed to PD, 20231005@njucm.edu.cn.
