# Supplementary material for: Development of machine learning models for predicting postoperative hyperglycemia in non-diabetic gastric cancer patients: a retrospective cohort study analysis
Source: Front Endocrinol (Lausanne). 2025 Nov 10;16:1687745. doi: 10.3389/fendo.2025.1687745 (PMC12640856; doi:10.3389/fendo.2025.1687745)
Supplement: Supplementary file 1 [file DataSheet1.pdf]

Supplementary Material

Development of Machine Learning Models for Predicting Postoperative Hyperglycemia in Non-Diabetic Gastric Cancer Patients: A Retrospective Cohort Study Analysis

Nan Wang[12#], Jie Zhang[12#], Chaonan Fei[3], Ye Ding[4], Li Yang[4], Peibei Duan[12*]

[1]School of Nursing, Nanjing University of Chinese Medicine, Nanjing, China

[2]Department of Nursing, Jiangsu Province Hospital of Chinese Medicine, Affiliated Hospital of Nanjing University of Chinese Medicine, Nanjing, China

[3]Department of Oncology, Jiangsu Province Hospital of Chinese Medicine, Affiliated Hospital of Nanjing University of Chinese Medicine, Nanjing, China

[4]Department of Endocrinology, Jiangsu Province Hospital of Chinese Medicine, Affiliated Hospital of Nanjing University of Chinese Medicine, Nanjing, China

[#]These authors contributed equally.

[*] Correspondence Author:

Peibei Duan, Nanjing University of Chinese Medicine No. 138 Xianlin Road, Qixia District, Nanjing 210023, China; Email: 20231005@njucm.edu.cn

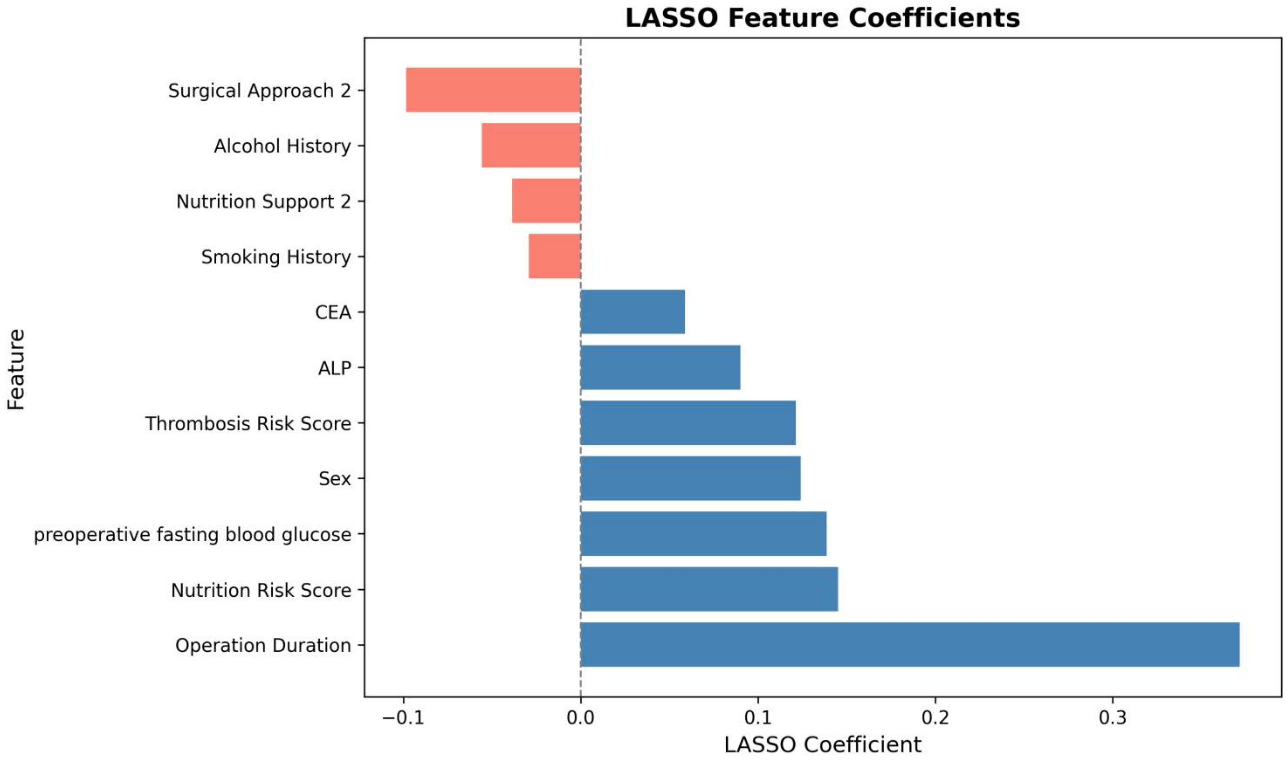

Supplementary Figure S1. LASSO coefficient plot for feature selection.

This plot illustrates the coefficients of variables retained by LASSO logistic regression with L1 regularization (C = 0.1). Positive and negative coefficients indicate features that increase or decrease the risk of postoperative hyperglycemia, respectively. Only variables with non-zero coefficients are displayed, providing a visual representation of each feature’ s relative contribution to the model, with Surgical Approach 2 representing robotic surgery.

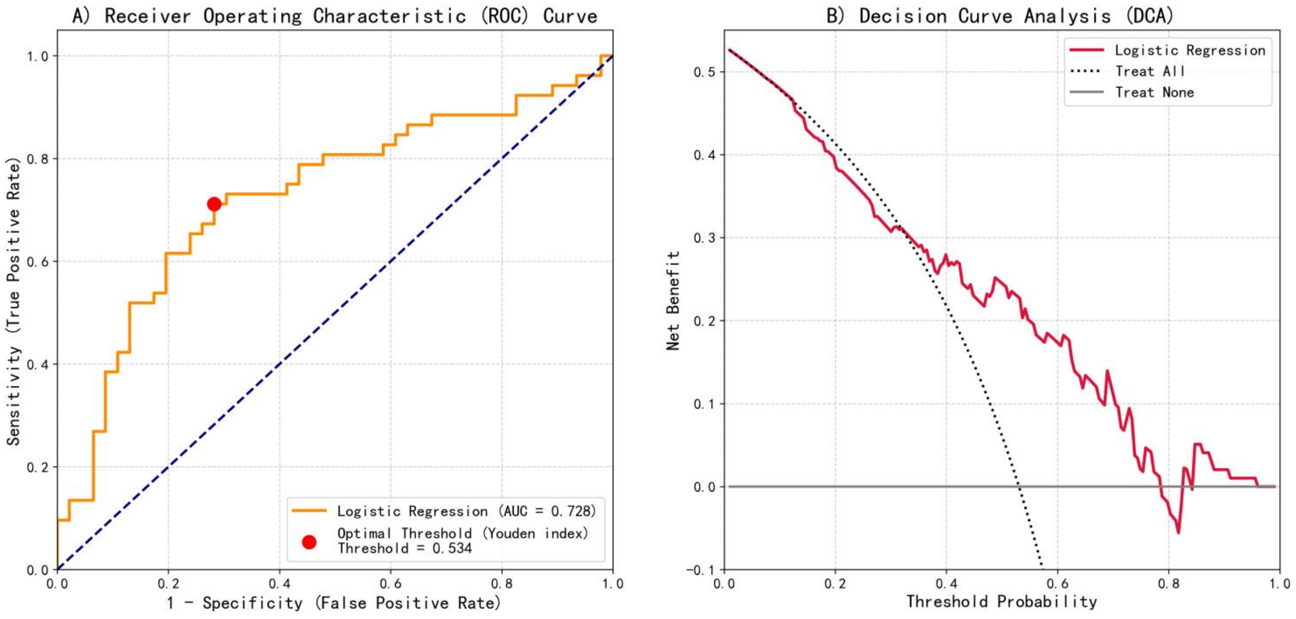

Supplementary Figure S2. ROC and decision curve analysis (DCA) of the nomogram model for predicting postoperative hyperglycemia.

A) Receiver operating characteristic (ROC) curve demonstrating the discriminative ability of the nomogram model. The area under the curve (AUC) is 0.728, and the optimal threshold based on the Youden index is indicated by the red dot.

B) Decision curve analysis (DCA) illustrating the clinical net benefit of the nomogram model across a range of threshold probabilities. The red line represents the nomogram, the dotted line represents a "treat-all" strategy, and the gray line represents a "treat-none" strategy. The nomogram provides greater net benefit than the “treat-all” and “treat-none” approaches, supporting its potential clinical utility.
